# Controlling process instability for defect lean metal additive manufacturing

**DOI:** 10.1038/s41467-022-28649-2

**Published:** 2022-02-28

**Authors:** Minglei Qu, Qilin Guo, Luis I. Escano, Ali Nabaa, S. Mohammad H. Hojjatzadeh, Zachary A. Young, Lianyi Chen

**Affiliations:** 1 Department of Mechanical Engineering, University of Wisconsin-Madison, Madison, WI 53706 USA; 2 Department of Materials Science and Engineering, University of Wisconsin-Madison, Madison, WI 53706 USA

**Keywords:** Mechanical engineering, Metals and alloys

## Abstract

The process instabilities intrinsic to the localized laser-powder bed interaction cause the formation of various defects in laser powder bed fusion (LPBF) additive manufacturing process. Particularly, the stochastic formation of large spatters leads to unpredictable defects in the as-printed parts. Here we report the elimination of large spatters through controlling laser-powder bed interaction instabilities by using nanoparticles. The elimination of large spatters results in 3D printing of defect lean sample with good consistency and enhanced properties. We reveal that two mechanisms work synergistically to eliminate all types of large spatters: (1) nanoparticle-enabled control of molten pool fluctuation eliminates the liquid breakup induced large spatters; (2) nanoparticle-enabled control of the liquid droplet coalescence eliminates liquid droplet colliding induced large spatters. The nanoparticle-enabled simultaneous stabilization of molten pool fluctuation and prevention of liquid droplet coalescence discovered here provide a potential way to achieve defect lean metal additive manufacturing.

## Abstract (web summary)

Defects induced by process instabilities in metal additive manufacturing limit its applications. Here, the authors report controlling laser-powder bed interaction instabilities by nanoparticles leads to defect lean metal additive manufacturing.

## Introduction

**Fig. 1:**
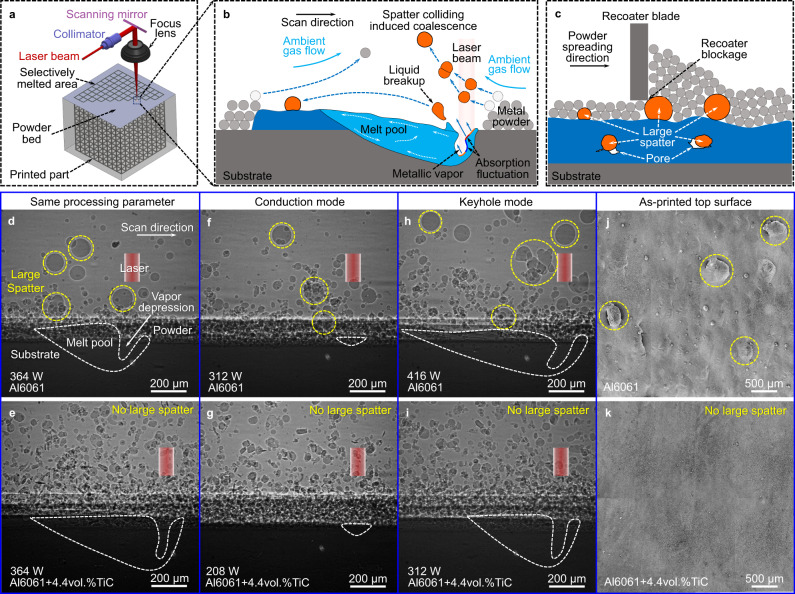
Nanoparticle-enabled elimination of large spatters in LPBF. **a** Focused laser beam enables LPBF to print complex geometries. **b** Intrinsic process instabilities of LPBF cause large spatter generation. **c** Defects induced by large spatters. **d**–**i** In situ x-ray images showing the spatter behavior comparison of Al6061 and Al6061+4.4vol.%TiC under the same processing parameter (**d**, **e**), and under the same melting mode, conduction mode (**f**, **g**) and keyhole mode (**h**, **i**). The laser power is indicated in the x-ray image. The scan speed is 0.4 m s^−1^. Large spatters are indicated by yellow dashed circles. **j**, **k** SEM images showing the top surfaces of the as-printed Al6061 (**j**) and Al6061+4.4vol.%TiC (**k**). Elimination of large spatters is observed. The in situ x-ray imaging videos corresponding to **d**–**i** are provided in Supplementary Movies 1–3.

Laser powder bed fusion (LPBF) uses a focused high-energy laser beam to selectively melt thin layers of metal powders to directly convert a computer-aided design model to a part^1^. The high spatial resolution stemming from the small focused beam size (about 50–100 µm) gives LPBF the capability to manufacture metal parts with complex geometries unachievable by conventional manufacturing routes^2^, as shown in Fig. 1a, which has the potential to revolutionize many industries (e.g., aerospace, medical, defense)^3–5^. However, the focused laser heating of a powder bed creates severe process instabilities, which causes the formation of various defects^6–11^, as schematically shown in Fig. 1b, c. As the high-energy laser beam impinges on the powder bed, the localized laser heating causes surface boiling to form a strong vapor jet^9^. The recoil pressure created by the vapor jet pushes the melt surface downward to form a vapor depression (also known as depression zone or keyhole)^12^; the high-speed upward vapor flow of the vapor jet ejects powders and liquid droplets away to form spatters and induces ambient gas flow toward the laser beam to cause powder entrainment.

Due to the strong dependence of the laser absorptivity on the incident angle^13^, the nonuniform energy absorption leads to nonuniform vaporization that causes nonuniform recoil pressure on the melt pool surface (liquid-gas interface). The nonuniform recoil pressure causes the fluctuation of the liquid-gas interface, which further causes the fluctuation of laser energy absorption and vapor pressure. The mutually supported energy absorption fluctuation and liquid surface fluctuation cause strong laser-powder bed interaction instabilities, e.g., melt pool/vapor depression fluctuation and gas-flow-driven spatter colliding. The vapor depression fluctuation induced liquid breakup^14^ from the melt pool and the gas-driven spatter colliding^15^ can cause the formation of large spatters (here defined as the spatter with the size larger than 100 µm).

The stochastic formation of large spatters is the major cause of unpredictable defect formation in the LPBF process and a big challenge for quality control^15–18^, because it could cause critical processing faults (e.g., recoater blockage^18^, powder bed non-uniformity^19^, surface pit^20^, balling^21^, melt track distortion^17^), and defects in the printed parts (e.g., lack of fusion porosity^7,22^, inclusion^23,24^). The part quality inconsistency induced by unpredictable defects is the most prominent barrier for the widespread adoption of the LPBF in various industries, especially for mission-critical applications^25,26^.

Previous efforts on optimizing processing conditions could alter the amount of spatter, but could not eliminate the large spatter, because tuning the processing parameter cannot change the intrinsic nature of the localized interaction of laser with powder bed^15^. Eliminating the stochastic formation of large spatters remains a challenge.

Here we show the elimination of large spatters by using nanoparticles to control the laser-powder bed interaction, which leads to 3D printing of defect lean samples with good consistency and enhanced properties. Our in situ high-speed synchrotron x-ray imaging experiments reveal that nanoparticles eliminate all types of large spatters by simultaneously stabilizing molten pool fluctuation and controlling liquid droplet coalescence. The method and mechanisms we discovered here provide a potential way to achieve defect lean metal additive manufacturing.

## Results and discussion

### Elimination of large spatters

We demonstrated the nanoparticle-enabled elimination of large spatters in Al6061+4.4vol.%TiC nanoparticles system (details about materials and sample preparation are described in Methods, Supplementary Notes 1 and 2, Supplementary Figs. 1 and 2, and Supplementary Table 1). In situ high-speed synchrotron x-ray imaging was used to characterize the spattering dynamics during laser melting of Al6061+4.4vol.%TiC powder bed, as well as Al6061 powder bed for comparison (see Methods for details). We first studied the spattering behavior under the same processing parameter. As shown in Fig. 1d, e and Supplementary Movie 1, many large spatters (with sizes larger than the layer thickness of 100 µm) were generated with a frequency of about 3 ± 1 (mean ± standard deviation) spatters per millisecond during laser melting of Al6061 powder bed; in sharp contrast, no large spatter was observed during laser melting of Al6061+4.4vol.%TiC powder bed. We noticed that the introduction of nanoparticles increases the vapor depression depth, which is mainly caused by the enhanced absorptivity by nanoparticles as shown in Supplementary Fig. 3, Supplementary Note 3, and references^27,28^. To confirm that the large spatter elimination is not caused by the change of the vapor depression depth and is not just limited to a certain processing parameter, we conducted in situ x-ray imaging experiments in a wide range of processing parameters. The nanoparticle-enabled elimination of large spatters was observed under all the processing conditions studied. Figure 1f–i and Supplementary Movies 2 and 3 show two more examples, which are comparisons of spattering behavior under the same melting mode with similar vapor depression depth.

The elimination of large spatters by nanoparticles was further confirmed by single layer printing experiments (see details in Methods). The scanning electron microscopy (SEM) images of the as-printed surface after removing the loose powders show the absence of large spatters on the surface of the Al6061+4.4vol.%TiC sample (Fig. 1k); in contrast, many large spatters with a number density of about 25 ± 4 cm^–2^ were observed on the surface of the Al6061 sample (Fig. 1j). The loose powders were collected and analyzed by sieving. All the loose powders for Al6061+4.4vol.%TiC sample went through the 100 µm sieve, indicating the complete elimination of large spatters by nanoparticles.

### Printing of defect lean sample with good consistency and enhanced properties

**Fig. 2:**
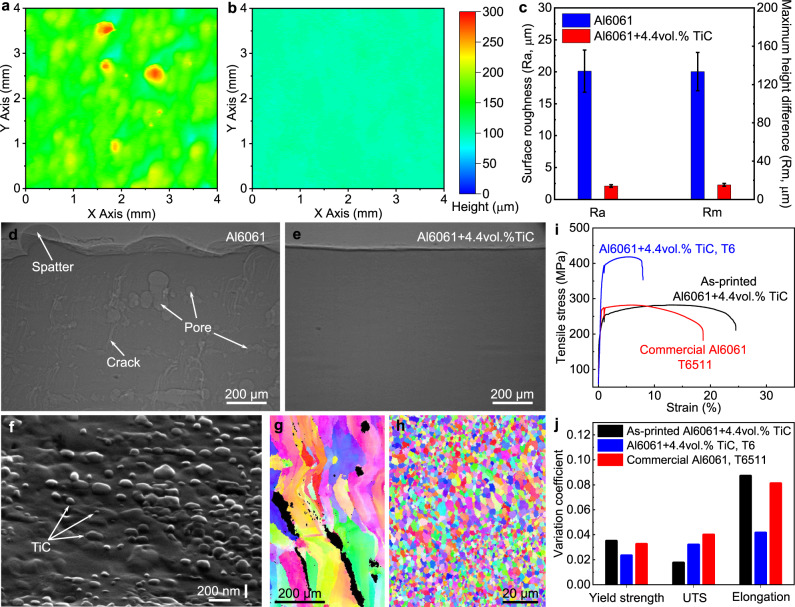
Microstructure and properties. **a**, **b** Surface profiles of the as-printed top surface of Al6061 (**a**) and Al6061+4.4vol.%TiC (**b**). **c** Surface roughness (Ra) and maximum height difference (Rm) of the as-printed top surface. The error bars represent the standard deviation. **d**, **e** X-ray images of as-printed Al6061 (**d**) and as-printed Al6061+4.4vol.%TiC (**e**) samples. **f** SEM image showing TiC nanoparticle distribution in the cross-section of the as-printed Al6061+4.4vol.%TiC. The sample is tilted 52° to show nanoparticles more clearly. **g**, **h** Inverse pole figure (IPF) images of the as-printed Al6061 (**g**) and the as-printed Al6061+4.4vol.%TiC (**h**). Black color in **g** represents cracks or pores. **i** Representative tensile test curves. The sudden drop of strength at the strain of 1% is caused by the extensometer removal. **j** The variation coefficient of yield strength, ultimate tensile strength (UTS), and elongation. The as-printed Al6061 sample was not tested because it contains too many cracks (as shown in **d**).

The elimination of large spatters leads to a dramatic reduction of defects in the printed sample. Surface profile measurements show that the surface roughness (Ra) was reduced by 90%, from 20 ± 3 µm in Al6061 to 2.1 ± 0.2 µm in Al6061+4.4vol.%TiC, and the maximum height difference (Rm, the height difference between the highest peak and lowest valley) was reduced by 89% from 134 ± 20 to 15 ± 2 µm (Fig. 2a–c). X-ray imaging inspections with a spatial resolution of 2 µm illustrate that no pore was detected in the as-printed Al6061+4.4vol.%TiC sample; however, multiple pores were observed in the as-printed Al6061, as shown in Fig. 2d, e and Supplementary Fig. 4. In addition, the dispersed nanoparticles in the Al6061 matrix, as shown in Fig. 2f, also refined the grain of the as-printed sample by more than one order of magnitude, from 66 ± 34 µm in Al6061 to 2 ± 0.2 µm in Al6061+4.4vol.%TiC (Fig. 2g, h). The effective grain refinement results in the elimination of hot cracking (Fig. 2d, e)^29,30^. A defect lean as-printed sample was successfully obtained.

Tensile testing results show that the as-printed Al6061+4.4vol.%TiC achieves a tensile strength comparable to, and a tensile elongation 33% higher than, that of the wrought Al6061 after T6 heat treatment; the heat-treated Al6061+4.4vol.%TiC exhibits a tensile strength of 123 ± 13 MPa (42%) higher than that of the wrought Al6061 and still maintains a reasonable elongation of 7.9% ± 0.3% (Fig. 2i). The wrought Al6061 is used for comparison because the as-printed Al6061 sample contains cracks (Fig. 2d, g). More importantly, the mechanical properties of the printed Al6061+4.4vol.%TiC exhibit good consistency (comparable to wrought Al6061), as quantified by the variation coefficient (standard deviation/average value) shown in Fig. 2j. Detailed data on tensile testing results are shown in Supplementary Fig. 5 and Supplementary Table 2.

### Mechanisms of nanoparticle-enabled elimination of liquid breakup induced spatters

To unravel the mechanisms underlying the nanoparticle-enabled large spatter elimination, we conducted an in-depth in situ high-speed x-ray imaging study. We discovered two mechanisms that work synergistically to prevent large spatter formation.

**Fig. 3:**
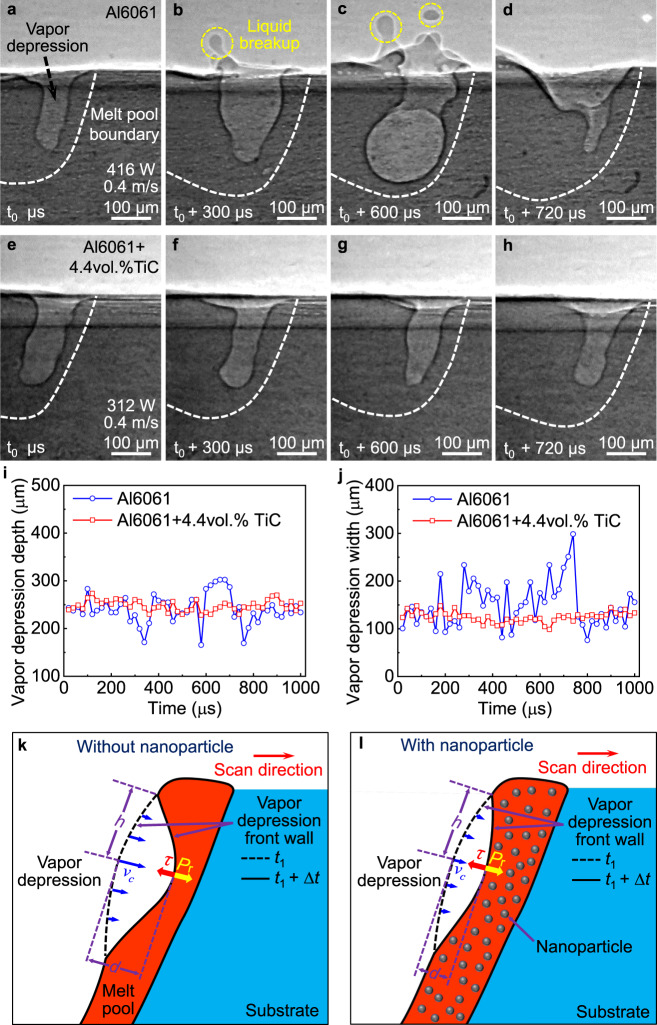
Nanoparticle-enabled elimination of liquid breakup. **a**–**d** X-ray images showing the liquid breakup during laser melting of Al6061. The liquid breakup is indicated by the yellow dashed circle. **e**–**h** X-ray images showing the more stable vapor depression without liquid breakup during laser melting of Al6061+4.4vol.%TiC. **i**, **j** The vapor depression depth and width evolution during laser melting. Since vapor depression depth plays a key role in determining spatter formation, the laser processing parameters were selected to achieve similar vapor depression depth in Al6061 and Al6061+4.4vol.%TiC for comparison. The comparison under the same laser processing parameter is shown in Supplementary Fig. 6. **k**, **l** Schematic of nanoparticle-enabled stabilization of the vapor depression fluctuation. The fluctuation of vapor depression creates a velocity gradient along the tangential direction of the vapor depression front wall, which results in the generation of viscous stress: *τ* = *μ*d*v*/d*x* = *μv*_*c*_/*h*, where *τ* is the viscous shear stress; *µ* is the viscosity; *v* is the moving velocity of vapor depression front wall induced by depression fluctuation, *x* is the distance along vapor depression front wall, ${v}_{{{\mbox{c}}}}$ is the front wall moving velocity at the center of the fluctuation, $h$ is the distance from center to the edge of the fluctuation. Due to the increased viscosity (*μ*) by nanoparticles, smaller front wall moving velocity (${v}_{{{\mbox{c}}}}$) is needed for generating the same viscous stress to resist recoil pressure (*P*_r_). Therefore, the depth of the fluctuation (*d* = *v*_*c*_Δ*t*, where Δ*t* is the time period and is considered constant to study the deformation within the same time period) decreased, resulting in the stabilization of vapor depression.

The first mechanism we found is that the nanoparticles stabilize the vapor depression fluctuation, which results in the elimination of the liquid breakup from the melt pool (Fig. 3 and Supplementary Fig. 6). In the reference sample Al6061, as expected, we observed the liquid breakup from the melt pool around the vapor depression rim to form spatters (Fig. 3a–d and Supplementary Movie 4). Simulation studies show that the liquid breakup is caused by the inertial pressure induced by the momentum of the fluid overcoming the capillary pressure induced by surface tension^9,16^, which is similar to the splash of water. However, in Al6061+4.4vol.%TiC, no liquid breakup (or even no liquid protrusion) was observed (Fig. 3e–h and Supplementary Movie 4). The bare substrate (sample without powder layer) was selected for this set of experiments to avoid the influence of powders on the liquid breakup observation.

The analysis of the fluctuation of the vapor depression depth and width shows that the absence of liquid breakup can be attributed to the stabilization of the vapor depression fluctuation (Fig. 3i, j). We hypothesize that the stabilization of the vapor depression fluctuation stems from the nanoparticle-induced increase of viscosity, as detailed in Supplementary Notes 4 and 5, Supplementary Figs. 7 and 8, Supplementary Tables 3 and 4, and schematically illustrated in Fig. 3k, l. Due to the increased viscosity, less liquid surface deformation is needed for generating viscous stress to balance the recoil pressure, which leads to less vapor depression fluctuation. With reduced vapor depression fluctuation, the driving force for the sudden increase of liquid momentum (e.g., sudden expansion of the vapor depression that pushes the liquid to move up^15^, bulk explosion that expels liquid out of the melt pool^31^) is mitigated. Without sufficient liquid momentum, the inertial pressure (i.e., kinetic energy) cannot overcome the surface tension induced capillary pressure to cause spattering.

### Mechanisms of nanoparticle-enabled elimination of droplet colliding induced large spatters

**Fig. 4:**
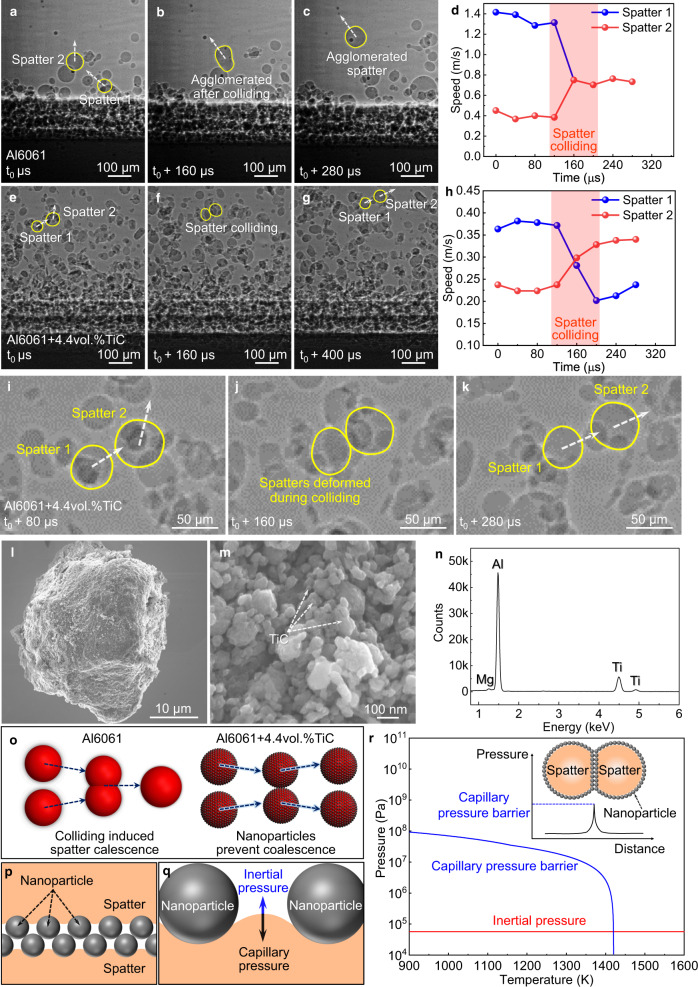
Nanoparticle-enabled prevention of spatter coalescence. **a**–**c** X-ray images showing the spatter colliding induced agglomeration during the LPBF of Al6061. **d** The moving speed of Al6061 spatters. **e**–**g** X-ray images showing that two Al6061+4.4vol.%TiC spatters separated after colliding. **h** The moving speed of Al6061+4.4vol.%TiC spatters. **i**–**k** X-ray images showing the deformation of Al6061+4.4vol.%TiC spatters during colliding. The moving direction of the spatter is indicated by the white dashed arrow. **l** SEM image of the ball-milled Al6061+4.4vol.%TiC powder. **m** SEM image of the surface of the powder in **l**. **n** EDS analysis of Al6061+4.4vol.%TiC powder surface. **o** Nanoparticles prevented coalescence during spatter colliding. The blue dashed arrows represent the spatter moving velocity. **p** Schematic illustration of nanoparticle arrangement between two colliding Al6061+4.4vol.%TiC spatters. **q** Schematic illustration showing nanoparticles on spatter surface create a capillary pressure. **r** Comparison of inertial pressure and capillary pressure barrier as a function of temperature. Inset: The capillary pressure barrier between two colliding Al6061+4.4vol.%TiC spatters.

The second mechanism we discovered is that the nanoparticles prevent the liquid spatter coalescence during colliding, which leads to the elimination of the colliding induced large spatters. Powder colliding happens frequently during LPBF process due to the intensive and chaotic gas flow around the laser-powder bed interaction area. As shown in Fig. 4a–d, Supplementary Fig. 9a–d, and Supplementary Movie 5, when two liquid spatters in Al6061 collide, the two spatters merge to form a large spatter. The colliding induced agglomeration is the major mechanism for large spatter formation, which is extremely difficult to overcome by optimizing processing condition or tuning alloy composition. However, we found that the liquid spatters can separate immediately after colliding during laser melting of Al6061+4.4vol.%TiC powder bed, as shown in Fig. 4e–k, Supplementary Fig. 9e–h, and Supplementary Movie 6. The two colliding spatters maintained their initial sizes; the only consequence of the colliding is the change of their moving directions and speeds (Fig. 4h and Supplementary Fig. 9h).

To find out the mechanisms of the coalescence prevention observed in Al6061+4.4vol.%TiC, we analyzed the feedstock powder surface by SEM. The results show that a layer of TiC nanoparticles covers the surface of the feedstock powders (Fig. 4l–n). We hypothesize that when the powder is melted, the nanoparticles on the surface of the liquid droplet will create a capillary pressure barrier against liquid droplet coalescence when they collide, as schematically illustrated in Fig. 4o–q and the inset of Fig. 4r. If assuming a closely packed single layer of nanoparticles on the surface of each colliding liquid spatter, the capillary pressure barrier, i.e., the maximum capillary pressure, can be estimated using the following equation^32^:

1 $${P}_{{{{{{\rm{barrier}}}}}}}=c\frac{2{\sigma }}{R}[z-{{{{{\rm{cos}}}}}}({\theta })]$$

where *σ* is the surface tension of liquid Al6061, $R$ is the radius of nanoparticle, *θ* is the contact angle of Al6061 on TiC, *c* is a constant related to coverage fraction of nanoparticles on spatter surface (the ratio of projection areas of the nanoparticles to the surface area of the spatter), *z* is a constant related to the number of nanoparticle layers between the two liquid spatter surfaces. For closely packed double-layer nanoparticles at the interface of the two colliding droplets (Fig. 4p), *c* is 2.73 for *θ* < 90°, 4.27 for *θ* > 90°. *z* is 0.633 for *θ* < 90°, 0.405 for *θ* > 90°. A guideline for estimating the volume fraction of nanoparticles needed to prevent droplet coalescence is presented in Supplementary Note 6 and Supplementary Fig. 10.

The calculated capillary pressure barrier using the temperature-dependent surface tension^33^ and contact angle^34^ indicates that the capillary pressure barrier can be two orders of magnitude higher than the estimated inertial pressure (i.e., kinetic energy that promotes direct liquid-liquid contact for coalescence, indicated by blue arrows in Fig. 4q, detailed in Supplementary Note 7) from melting temperature to 526 K above the melting temperature of Al6061. This high-pressure barrier is sufficient to prevent liquid spatter coalescence during colliding.

Since the capillary pressure barrier depends on the temperature and nanoparticle packing (as shown in Fig. 4p–r and Supplementary Fig. 10), if the temperature of the spatter is too high (e.g., higher than 1420 K in Al6061-TiC system) or the nanoparticle on the surface of the spatter is not enough, the coalescence will still happen. This speculation is supported by our coalescence frequency statistical analysis results: even though the spatter coalescence frequency decreases significantly with the addition of 4.4vol.%TiC (from 9 ± 2 per millisecond for Al6061 to 1 ± 0.1 per millisecond for Al6061+4.4vol.%TiC), spatter coalescence was still observed in Al6061+4.4vol.%TiC.

The occasional spatter coalescence in Al6061+4.4vol.%TiC does not lead to the formation of a large spatter (with a size larger than 100 µm) because the formation of the large spatter may need coalescence of multiple spatters. Even though the very hot spatters with an initial temperature higher than 1420 K can coalesce during the first colliding, they may cool down to below 1420 K to avoid the coalescence in the subsequent colliding.

The reduction of capillary pressure barrier at high temperature is critical for powder incorporation into the melt pool to form a build track. In the laser interaction zone, the powders can be heated to a very high temperature. This high temperature could eliminate the capillary pressure barrier in the melt pool region to allow the incorporation of nanoparticle-coated powders to enter the melt pool. We hypothesize that the temperature-dependent capillary pressure barrier is crucial for enabling spatter control and allowing powder incorporation into the melt pool at the same time.

In summary, we discover and demonstrate the control of laser-powder bed interaction instabilities by nanoparticles through simultaneously stabilizing molten pool fluctuation and preventing liquid droplet coalescence, which leads to the elimination of large spatters and printing of defect lean samples with good consistency and enhanced properties. Nanoparticle-enabled control of laser-matter interaction instabilities provides a viable path for achieving defect lean metal additive manufacturing.

## Methods

### Materials and sample preparation

The Al6061 powders (17–60 µm) were purchased from Valimet (USA). The Al6061 substrate (for x-ray imaging experiment with powder layer) and bare substrate (for x-ray imaging experiment without powder layer) were cut from the commercial Al6061 plate (T6511, Mcmaster-Carr, USA). The Al6061+TiC powders were prepared by planetary ball milling of Al6061 powders and TiC nanoparticles (83 nm, SSNano, USA). The Al6061+TiC substrate (for x-ray imaging experiment with powder layer) and bare substrate (for x-ray imaging experiment without powder layer) were cut from the as-printed Al6061+TiC samples. The as-printed Al6061+TiC samples were fabricated by LPBF of the ball-milled Al6061+TiC powders. During LPBF, a self-designed LPBF system was utilized, which includes a continuous-wave (CW) ytterbium fiber laser (IPG YLR-500-AC, IPG Photonics, USA), a galvo scanner (hurrySCAN 30, SCANLAB GmbH., Germany), and an experimental chamber filled with argon gas. The self-designed LPBF system was used because it can handle the non-spherical powders with low flowability (e.g., ball-milled powders). More detailed information about powder morphology, material composition, and sample preparation procedure is shown in Supplementary Figs. 1 and 2 and Supplementary Notes 1 and 2.

### High-speed x-ray imaging

High-speed high-resolution x-ray imaging (Beamline 32-ID-B, Advanced Photon Source, Argonne National Laboratory) was performed to capture the spatter, vapor depression and melt pool dynamics during laser scanning^35,36^. A CW ytterbium fiber laser (IPG YLR-500-AC, IPG Photonics, USA) and a galvo scanner (IntelliSCAN 30, SCANLAB GmbH., Germany) were integrated to perform single track laser melting experiment (laser beam size (D4σ) of 90 µm, laser power of 208–416 W, scan speed of 0.4 m s^−1^). During laser scanning, the synchrotron x-ray beam (exposure time of 1 µs) penetrated through the samples. The transmitted signal was captured by a detection system at 25 kHz for characterizing spattering behavior to acquire a larger field of view, and at 50 kHz for capturing vapor depression dynamics.

### High-speed visible light imaging of spatter dynamics

A high-speed visible light camera (FASTCAM Nova S12, Photron, Japan) with microscope optics (Infinity K2 DistaMax with CF-4 objective, Infinity Photo-Optical, USA) was used to capture the spatter dynamics during LPBF process from the top view. A CW ytterbium fiber laser (IPG YLR-500-AC, IPG Photonics, USA) and a galvo scanner (hurrySCAN 30, SCANLAB GmbH., Germany) were integrated to perform single track LPBF experiment (laser beam size (D4σ) of around 250 µm, laser power of 500 W, scan speed of 0.2 m s^−1^). The powder layer thickness is 100 µm. Imaging was performed at 25 kHz frame rate with an exposure time of 4 μs. The resolution is 4 μm. The view angle is 15° away from the normal direction of the powder bed surface. SugarCUBE Ultra illumination system (White LED Light SugarCUBE Ultra, Ushio, Japan) was used to illuminate the powder bed during the imaging experiment.

### Microstructure characterization

The nanoparticles in the as-printed Al6061+4.4vol.%TiC were characterized by SEM (Helios PFIB G4, FEI, USA). To reveal nanoparticles clearly, the sample was tilted by 52°. Before SEM, the polished samples were firstly cleaned by low-angle ion milling (10°, Fischione 1050 Ion Mill, E.A. Fischione Instruments, USA) and then slightly etched by gallium ions (ion beam imaging mode, 30 kV, 4 nA, Helios PFIB G4). Electron backscatter diffraction (EBSD, EDAX Hikari Super camera) analysis was performed to characterize the size, morphology and orientation of grains in the as-printed Al6061 and as-printed Al6061+4.4vol.%TiC with the step size of 3 and 0.2 µm, respectively. The artifacts at the grain boundary (typically 1 pixel) were cleaned by a grain dilation cleanup process using OIM analysis software. SEM (Zeiss 1530, ZEISS microscope, USA) was utilized to characterize the distribution of TiC nanoparticle on the surface of the ball-milled Al6061+4.4vol.%TiC powder.

### Tensile test

Tensile tests were performed using an Instron universal testing machine (Instron 5969, Instron, USA) with a strain rate of 2.5 × 10^−4^ s^−1^. At the beginning of the test, the extensometer (25.4 mm, attached to the specimen clamp) was used to measure/control strain. At a strain of 1%, the extensometer was removed, and the strain rate was further read out/controlled by the crosshead. The tensile specimen with a size of 9.5 mm (length) × 4.1 mm (width) × 1 mm (thickness) and a gauge section of 3 mm (length) × 1 mm (width) × 1 mm (thickness) developed for additively manufactured metal in reference^37^ (MT2) was used.

### Heat treatment

The T6 condition heat treatment on Al6061+4.4vol.%TiC was conducted using a muffle furnace under argon protected environment. The samples were first solutionized at 530 °C for 1 h, then quenched with water at 15 °C, finally aged at 160 °C for 18 h.

### Surface tension and viscosity measurement

The surface tension and viscosity were measured based on the oscillating droplet method^38^, as schematically shown in Supplementary Fig. 7a. During the test, the sample (cut from commercial Al6061 plate or as-printed Al6061+TiC sample) was placed on top of an inert ring (which holds the droplet) with an inner diameter of 2 mm and thickness of 0.5 mm. A CW ytterbium fiber laser (IPG YLR-500-AC, IPG Photonics, USA) was used to melt the sample with a weight of 1.3 × 10^−2^ g corresponding to a sphere with a diameter of 2.2 mm using a laser power of 150 W, laser beam size (D4σ) of 250 µm, and heating time of 2 s. After melting, the linear solenoid was immediately triggered to accelerate the ring, which forces the liquid droplet to flow through the ring and introduces the initial deformation. The oscillation of the droplet after leaving the ring was captured by a high-speed visible light camera (FASTCAM Nova S12, Photron, Japan) at a frame rate of 10 kHz. The frequency and amplitude of the oscillation are used to calculate the surface tension and viscosity, respectively, as detailed in Supplementary Note 4. The vacuum chamber was vacuumed and refilled with Argon gas three times before the experiment to create the same environment as the LPBF experiment.

## Supplementary information

Supplementary Information

Description of Additional Supplementary Files

Supplementary Movie 1

Supplementary Movie 2

Supplementary Movie 3

Supplementary Movie 4

Supplementary Movie 5

Supplementary Movie 6

## Data Availability

The data supporting the findings of this work are available in the main text or Supplementary Materials. Raw data are available from the corresponding author on reasonable request.
