# Publisher Correction: Controlling process instability for defect lean metal additive manufacturing

**DOI:** 10.1038/s41467-022-29295-4

**Published:** 2022-03-17

**Authors:** Minglei Qu, Qilin Guo, Luis I. Escano, Ali Nabaa, S. Mohammad H. Hojjatzadeh, Zachary A. Young, Lianyi Chen

**Affiliations:** 1 Department of Mechanical Engineering, University of Wisconsin-Madison, Madison, WI 53706 USA; 2 Department of Materials Science and Engineering, University of Wisconsin-Madison, Madison, WI 53706 USA

**Keywords:** Mechanical engineering, Metals and alloys

Correction to: *Nature Communications* 10.1038/s41467-022-28649-2, published online 28 February 2022.

The original version of this Article contained an error in Figure 4o, where the red spheres and black dots that represent spatters and nanoparticles, respectively, were missing. This has been corrected in both the PDF and HTML versions of the Article.

