# Supplementary material for: Controlling process instability for defect lean metal additive manufacturing
Source: Nat Commun. 2022 Feb 28;13:1079. doi: 10.1038/s41467-022-28649-2 (PMC8885710; doi:10.1038/s41467-022-28649-2)
Supplement: Supplementary file 1 — Supplementary Information [file 41467_2022_28649_MOESM1_ESM.pdf]

Supplementary Information

Controlling process instability for defect lean metal additive manufacturing

Minglei Qu[1,2], Qilin Guo[1,2], Luis I. Escano[1,2], Ali Nabaa[1,2], S. Mohammad H. Hojjatzadeh[1,2], Zachary A. Young[1], Lianyi Chen[1,2,*]

[1]Department of Mechanical Engineering, University of Wisconsin-Madison, Madison, Wisconsin 53706, USA.

[2]Department of Materials Science and Engineering, University of Wisconsin-Madison, Madison, Wisconsin 53706, USA.

[*]Correspondence to: Lianyi Chen (lianyi.chen@wisc.edu).

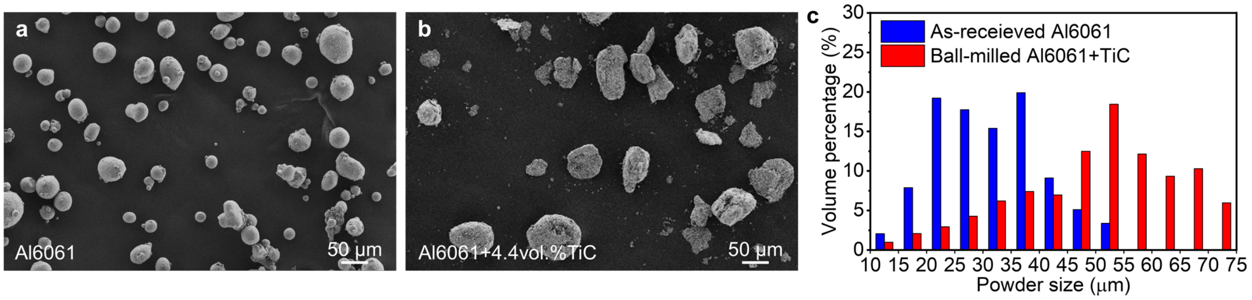

Supplementary Figure 1. The morphology and size distribution of powders. a, b, SEM images showing the morphology of the as-received Al6061 powder (**a**) and ball-milled Al6061+4.4vol.%TiC powder (**b**). **c,** The powder size distribution of the as-received Al6061 and ball-milled Al6061+4.4vol.%TiC powders.

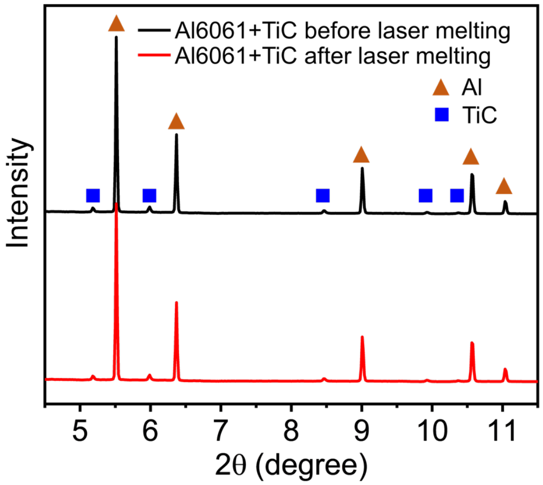

Supplementary Figure 2. XRD analysis of Al6061+TiC before and after laser melting. The black curve represents the XRD analysis result for Al6061+TiC before laser melting. The red curve represents the XRD analysis result for Al6061+TiC after laser melting. The consistent peaks of Al6061+TiC before and after laser melting in XRD analysis suggest that the reaction of TiC nanoparticles with Al6061 is negligible during laser melting process.

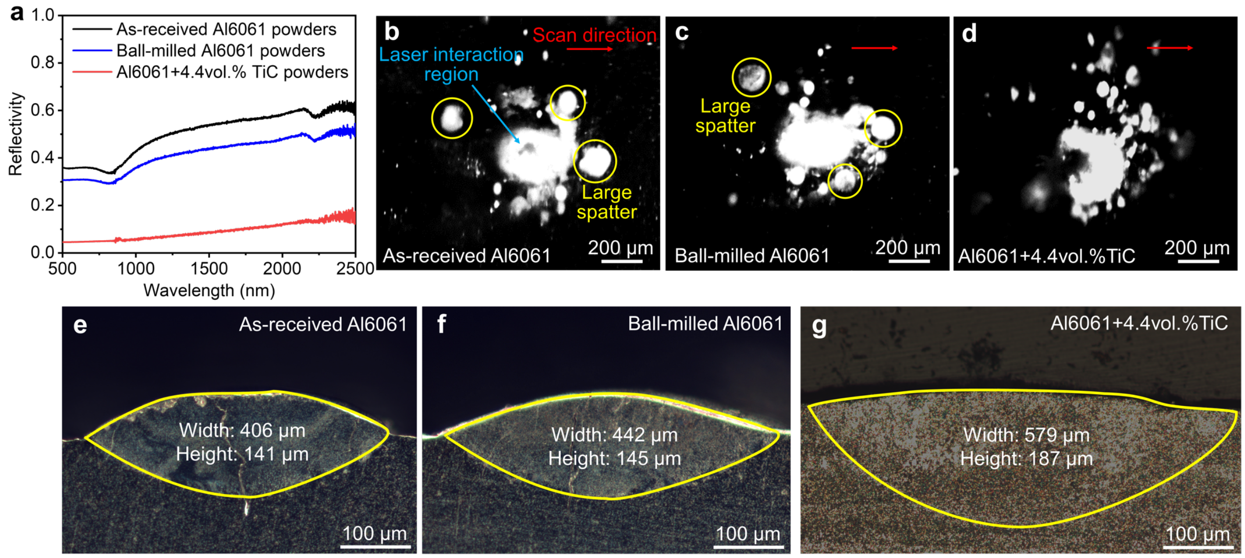

Supplementary Figure 3. Effects of powder morphology on laser-powder interaction. a, Diffuse reflectivity of as-received Al6061 powders, ball-milled Al6061 powders, and ball-milled Al6061+4.4vol.%TiC powders. **b-d,** High-speed visible light images showing spatter generation during LPBF of as-received Al6061 (with commercial Al6061 substrate) **(b)**, ball-milled Al6061 (with commercial Al6061 substrate) **(c)**, Al6061+4.4vol.%TiC (with as-printed Al6061+4.4vol.%TiC substrate) **(d)**. Large spatters were generated during LPBF of as-received Al6061 and ball-milled Al6061, while there is no large spatter during LPBF of Al6061+4.4vol.%TiC. The viewing angle of the camera is 15 degree away from the normal direction of the powder bed surface. **e-g,** Optical images showing the melt pool dimension (transverse cross section) of as-received Al6061 **(e)**, ball-milled Al6061 **(f)**, Al6061+4.4vol.%TiC **(g)** samples processed by LPBF. In **b-g**, the laser power is 500 W. The scan speed is 0.2 m s^{-1}. The laser beam diameter (D4σ) is around 250 μm.

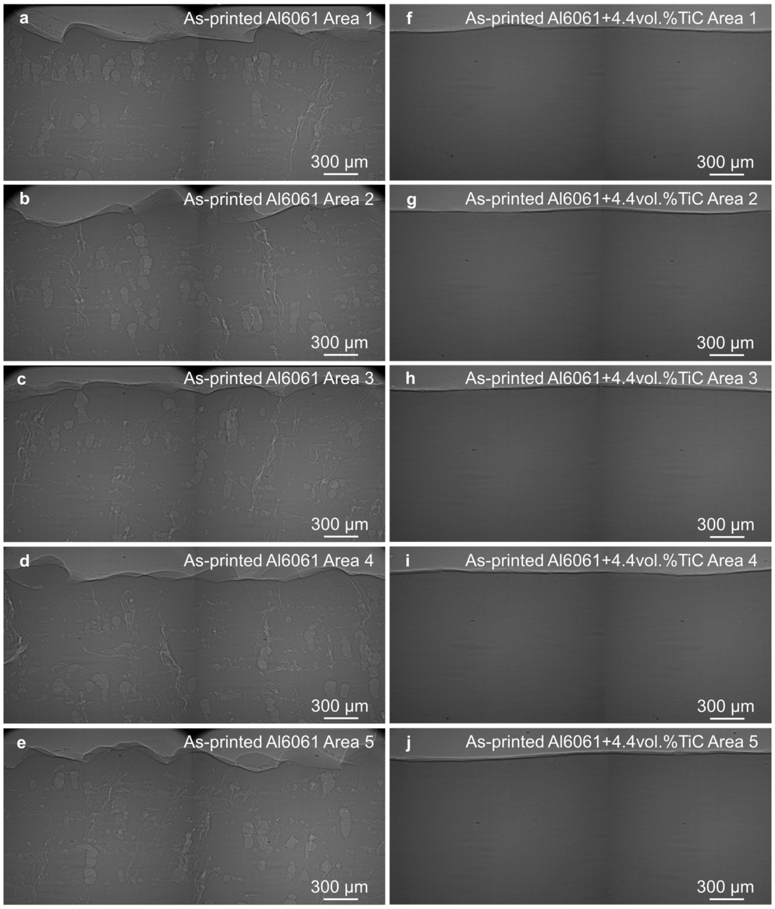

Supplementary Figure 4. X-ray images showing other areas of the as-printed Al6061 and as-printed Al6061+4.4vol.%TiC samples. a-e, X-ray images showing five other areas of as-printed Al6061 samples. **f-j,** X-ray images showing five other areas of as-printed Al6061+4.4vol.%TiC samples.

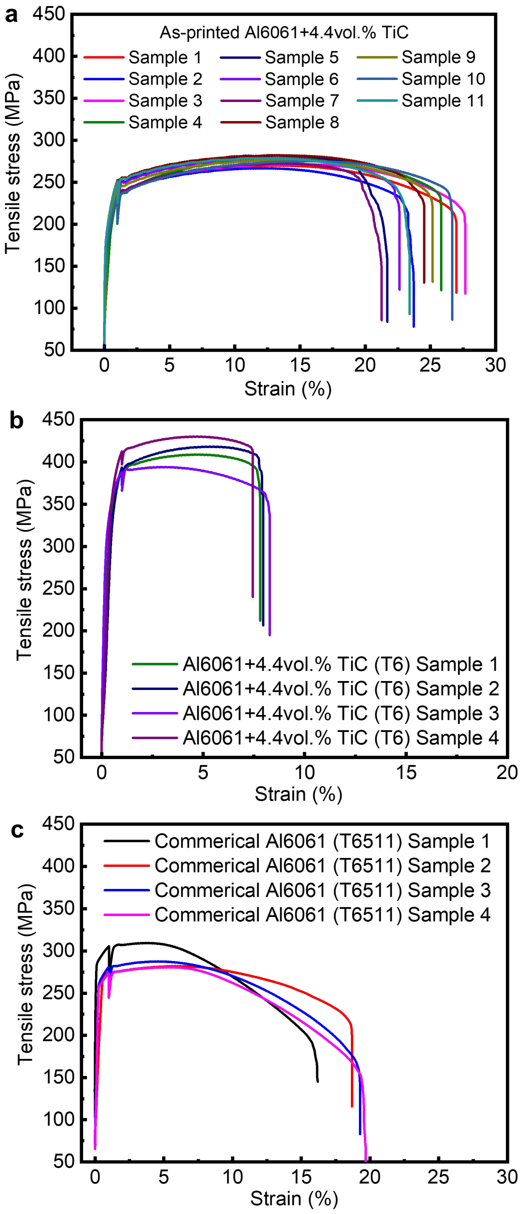

Supplementary Figure 5. Tensile test results. a-c, Tensile engineering stress-engineering strain curves of 11 as-printed Al6061+4.4vol%TiC samples **(a)**, four T6 heat-treated Al6061+4.4vol%TiC samples **(b)**, and four commercial Al6061 samples **(c)**. The sudden drop of stress at the strain of 1% is caused by the extensometer removing.

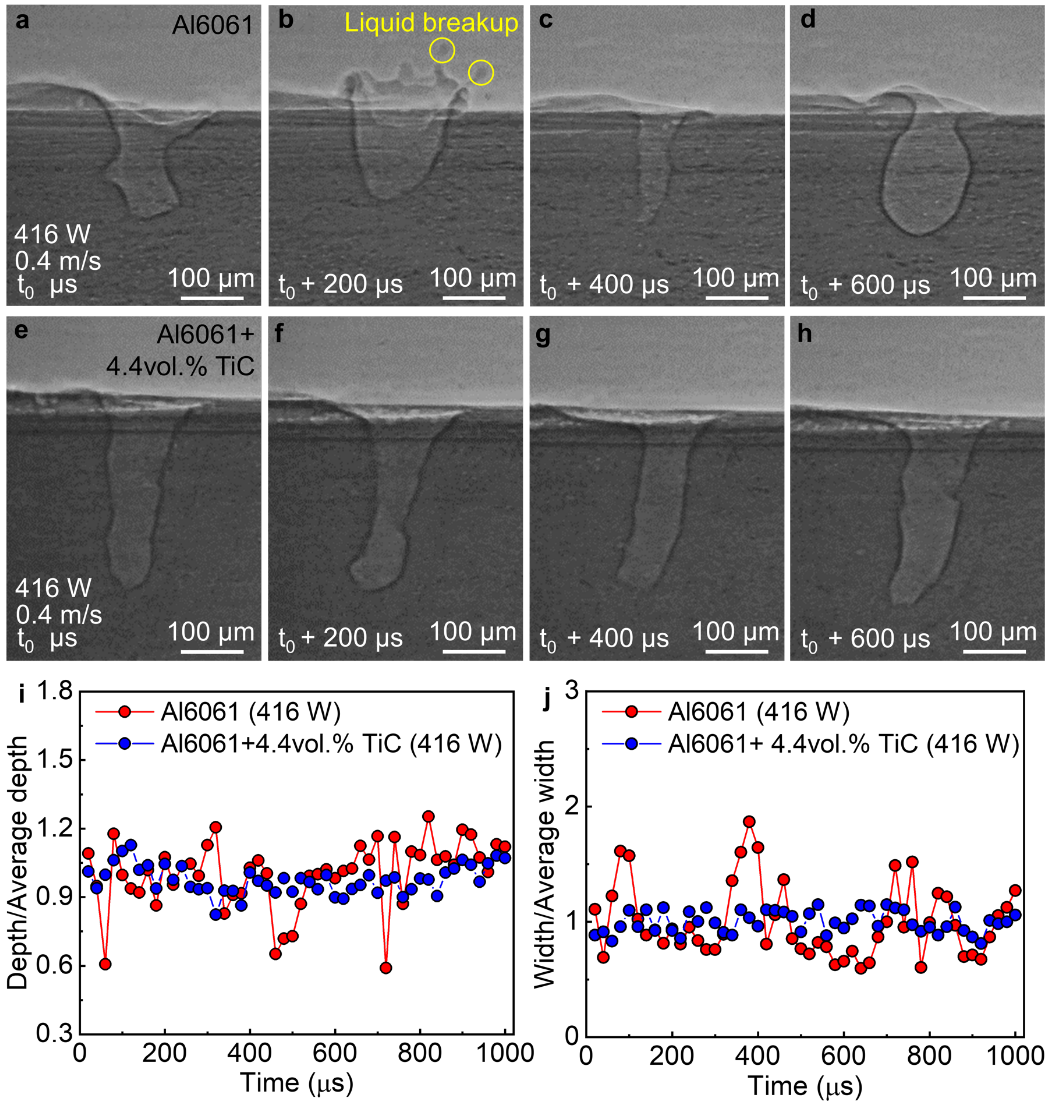

Supplementary Figure 6. X-ray images showing nanoparticles stabilize vapor depression and eliminate liquid breakup under the same processing parameter. a-h, X-ray images showing the vapor depression dynamics during laser melting of Al6061 **(a-d)** and Al6061+4.4vol.%TiC **(e-h)** bare substrates under the same laser processing parameter (laser power of 416 W, scan speed of 0.4 m s^{-1}, laser beam diameter (D4σ) of 90 μm). After adding TiC nanoparticles, the vapor depression is more stable, and the liquid breakup is eliminated. **i-j,** The relative fluctuation (dynamic dimension/average dimension) of vapor depression dimension during laser melting of Al6061 and Al6061+4.4vol.%TiC. Bare substrates were used for these experiments to avoid the influence of powders on the observation of liquid breakup.

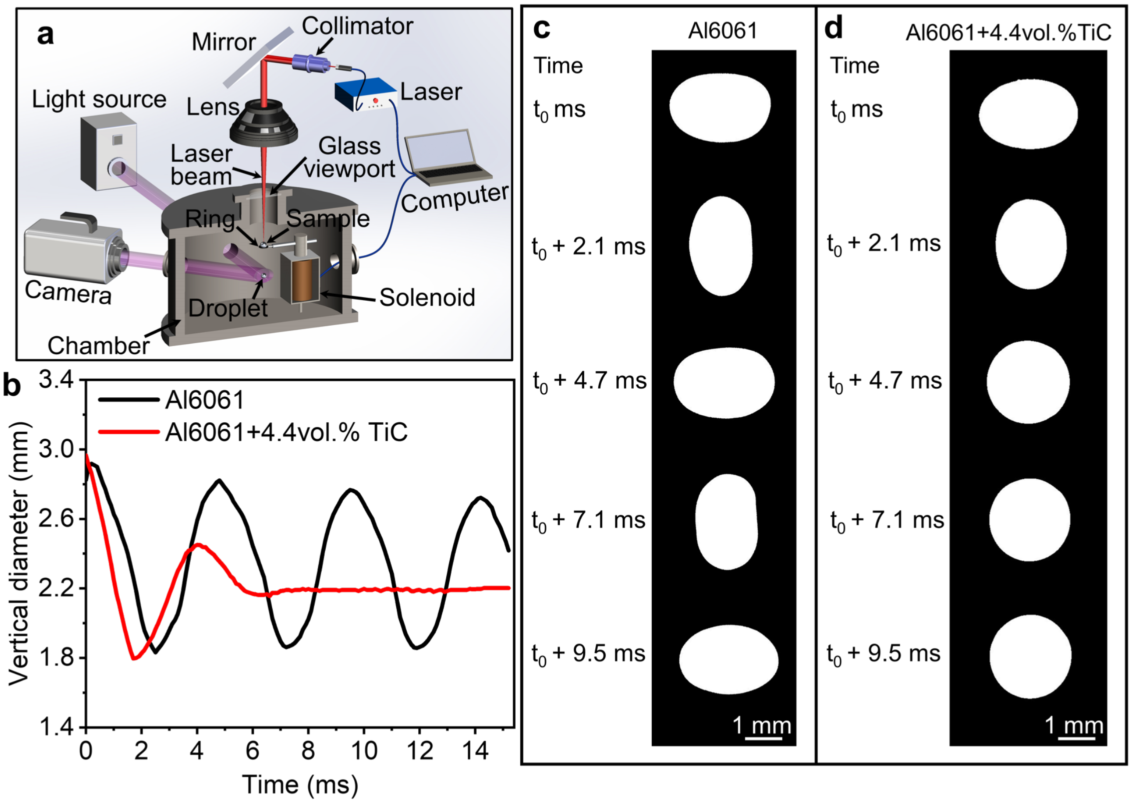

Supplementary Figure 7. Surface tension and viscosity measurement. a, Schematic showing the experimental setup. **b,** Vertical diameter of the liquid droplet as a function of time during droplet oscillation. **c, d,** High-speed visible light images showing the dynamic droplet morphology of Al6061 **(c)** and Al6061+4.4vol.%TiC **(d)** during oscillation.

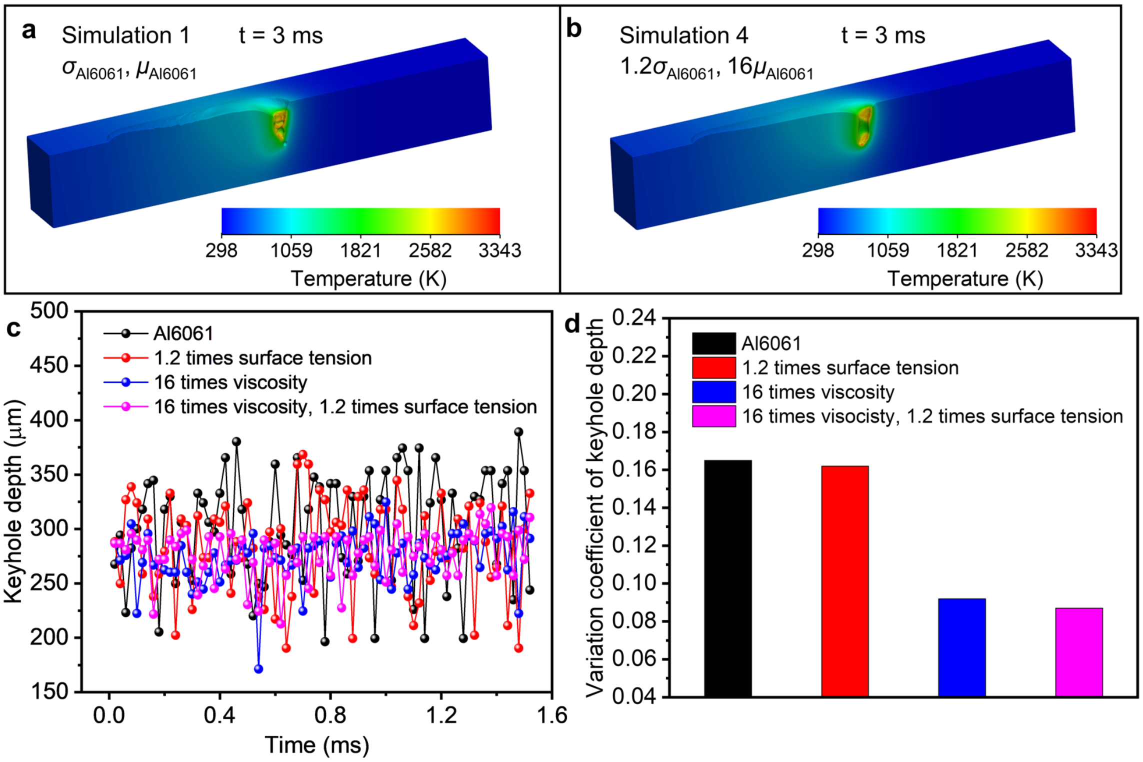

Supplementary Figure 8. Simulation of the effects of surface tension and viscosity on vapor depression fluctuation. a, b, Vapor depression morphology in laser melting simulation (Simulation 1 and Simulation 4 as defined in Supplementary Table 3) with different surface tension and viscosity values. The color scale is adjusted to highlight the vapor depression area. **c,** Dynamic vapor depression depth during laser melting process. **d,** Variation coefficient of vapor depression depth. In **c-d,** the vapor depression depth analysis was based on the simulation result.

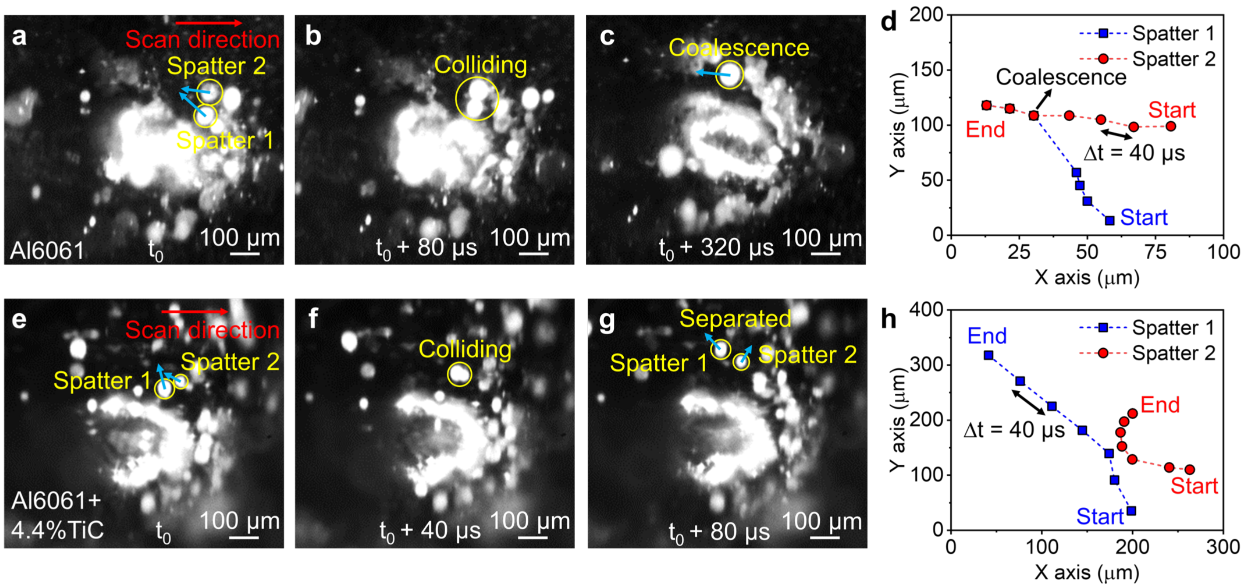

Supplementary Figure 9. High-speed visible light images showing nanoparticle-enabled prevention of spatter coalescence during colliding. a-c, Top-view high-speed visible light images showing two Al6061 spatters coalesced after colliding. **d,** Moving trajectory of two Al6061 spatters. **e-g,** Top-view high-speed visible light images showing two Al6061+4.4vol.%TiC spatters separated after colliding. **h,** Moving trajectory of two Al6061+4.4vol.%TiC spatters. Yellow circles indicate spatter. Blue arrows indicate spatter flying direction. The viewing angle of the camera is 15 degrees away from the normal direction of the powder bed surface. The laser power is 500 W. The scan speed is 0.2 m s^{-1}. The laser beam diameter (D4σ) is around 250 μm.

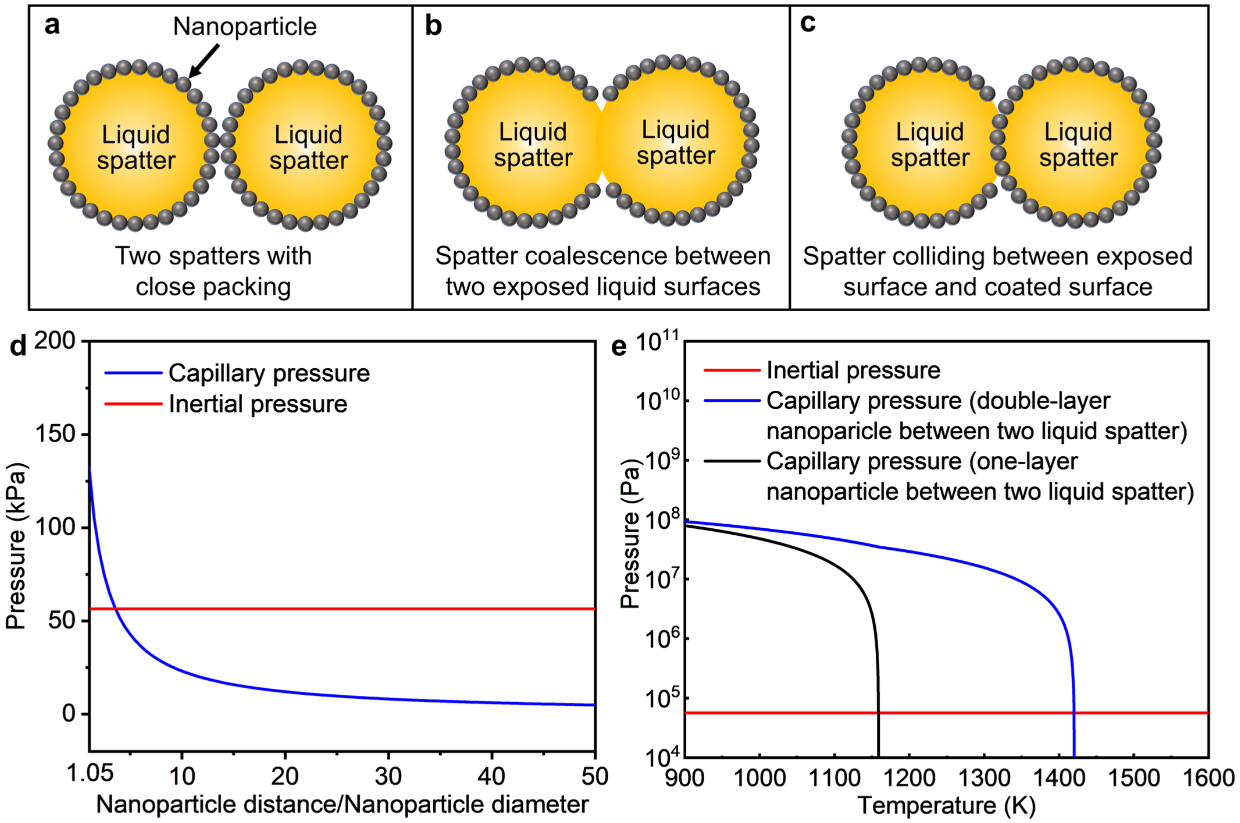

Supplementary Figure 10. The packing requirement of nanoparticles on spatter surface for controlling spatter coalescence. a, Schematic showing that close packing of nanoparticles on spatter surface prevents coalescence. **b,** Schematic showing that the colliding between two exposed surfaces of liquid spatters induces coalescence. **c,** Schematic showing the colliding between exposed spatter surface and coated spatter surface. **d,** The effect of interparticle spacing on capillary pressure barrier. The nanoparticle distance in **d** is defined as the center-to-center distance of two nearest nanoparticles on the spatter surface. **e,** The effect of number of nanoparticle layers between two spatters on capillary pressure barrier.

Supplementary Table 1. Chemical composition of as-received Al6061 powders, as-received Al6061 plate, and as-printed Al6061+4.4vol.%TiC.

Element (weight %)	Al	Cr	Cu	Fe	Mg	Mn	Si	Ti	Zn	Other
Al6061 powder, specification from Valimet	Balance	0.1	0.27	0.09	0.86	<0.01	0.55	0.01	<0.01	<0.15
Al6061 plate, specification from Mcmaster	Balance	0.2	0.28	0.4	0.96	0.12	0.69	0.02	0.01	<0.15
Al6061+4.4vol.%TiC, ICP test	Balance	0.1	0.27	0.14	0.44	0.004	0.52	6.3	0.001	<0.1

Supplementary Table 2. The summarized tensile test results.

Material	Yield strength (MPa)	Ultimate tensile strength (MPa)	Elongation
As-printed Al6061+4.4vol.%TiC	219 ± 8	276 ± 5	24.6% ± 2.1%
Al6061+4.4vol.%TiC, T6	389 ± 9	413 ± 13	7.9% ± 0.3%
Commercial Al6061, T6511	274 ± 9	290 ± 12	18.5% ± 1.5%

Note: Numbers represent average value ± standard deviation.

Supplementary Table 3. Surface tension and viscosity values used in the simulation. The number of 1.2 and 16 times are determined based on the measured surface tension and viscosity of Al6061+4.4vol.%TiC.The values of σ_{Al6061}, μ_{Al6061} are in **Supplementary Table 4**.

Simulation No.	1	2	3	4
Surface tension	σ_{Al6061}	$1.2\sigma_{Al6061}$	σ_{Al6061}	$1.2\sigma_{Al6061}$
Viscosity	μ_{Al6061}	μ_{Al6061}	$16\mu_{Al6061}$	$16\mu_{Al6061}$
Variation coefficient of keyhole depth	0.165	0.162	0.092	0.087

Supplementary Table 4. The material properties of Al6061 used in simulation.

Property	Value	
Solidus temperature (K)	873.15	
Liquidus temperature (K)	915.15	
Boiling temperature (K)	2792	
Solid density (kg m^{-3})	2705-0.201×(T-298)	
Liquid density (kg m^{-3})	2415-0.28×(T-915)	
Specific heat (J kg^{-1} K^{-1})	Temperature (T)	Value
	298.15	870
	373.15	950
	473.15	980
	573.15	1020
	673.15	1060
	773.15	1150
	873.15	1160
	915.15	1170
	973.15	1170
	1073.15	1170
Latent heat of melting (J kg^{-1})	336×10^3	
Surface tension at liquidus temperature (N m^{-1})	0.68†	
Surface tension coefficient (N m^{-1} K^{-1})	-0.155×10^{-3}	
Viscosity (mPa s)	Temperature (T)	Value
	915.15	4.9†
	973.15	4.5††
	1073.15	4.3††
Latent heat of vaporization (J kg^{-1})	10519×10^3	
Thermal conductivity (W m^{-1} K^{-1})	Temperature (T)	Value
	373.15	195
	473.15	203
	573.15	211
	673.15	212
	773.15	225
	873.15	200
	915.15	90
	973.15	91
	1073.15	92

Note:
The unit of the temperature (T) in the table is Kelvin (K).
† Measured in this work.
†† Estimated based on the measured value at 915.15 K and temperature dependence relation in Reference[1]

Supplementary Note 1: Materials and sample preparation

The Al6061 powders (17-60 μm) were purchased from Valimet (USA). The Al6061 substrate (for x-ray imaging experiment with powder layer) and bare substrate (for x-ray imaging experiment without powder layer) were cut from commercial Al6061 plate (T6511, Mcmaster-Carr, USA). Detailed information about powder size distribution and material composition is shown in Supplementary Fig. 1, and Supplementary Table 1, respectively. The Al6061+TiC powders were prepared by planetary ball milling of Al6061 powders and TiC nanoparticles (83 nm, SSNano, USA). The Al6061+TiC substrate (for x-ray imaging experiment with powder layer) and bare substrate (for x-ray imaging experiment without powder layer) were cut from the as-printed Al6061+TiC samples. The as-printed Al6061+TiC samples were fabricated by LPBF of the ball-milled Al6061+TiC powders. The LPBF system utilized includes a CW ytterbium fiber laser (IPG YLR-500-AC, IPG Photonics, USA), a galvo scanner (hurrySCAN 30, SCANLAB GmbH., Germany), and an experimental chamber filled with Argon gas. During printing, the laser power of 400 W and scan speed of 0.2 m s^{-1} were used. The laser beam size (D4σ) is around 250 μm. The hatch spacing is 80 μm. The nominal layer thickness is 50 μm. Unidirectional scan with 90° rotation scan vector between layers was employed as scan strategy. The printed area was 25 mm × 25 mm. The tensile specimen of Al6061+TiC was cut from the as-printed Al6061+TiC sample by wire electrical discharge machining (EDM). The tensile specimen with size of 9.5 mm (length) × 4.1 mm (width) × 1 mm (thickness) and a gauge section of 3 mm (length) × 1 mm (width) × 1 mm (thickness) developed for additively manufactured metal (MT2) in reference[2] was used in the tensile test. Smaller samples (than ASTM E8 standard) were used because (1) smaller sample enables us to study the effect of printing position on property variation within one build; (2) the printed area is relatively small (25 mm × 25 mm) for our self-designed LPBF system. To make the

tensile test results comparable, we also used the same sample size for tensile testing of the commercial Al6061.

Supplementary Note 2: Estimation of the volume fraction of TiC

Since TiC is relatively stable in the Al6061 melt with negligible reaction[3,4] (which is confirmed by XRD results in the Supplementary Fig. 2) and the Ti content in Al6061 powder is negligible, the volume fraction of TiC nanoparticles (V_{TiC}) was estimated based on the weight fraction of Ti in the as-printed sample by the following equation:

$$V_{\mathrm{TiC}} = \frac{W_{\mathrm{TiC}}\rho_{\mathrm{nc}}}{\rho_{\mathrm{TiC}}} = \frac{1.25W_{\mathrm{Ti}}\rho_{\mathrm{nc}}}{\rho_{\mathrm{TiC}}} \quad (1)$$

where W_{TiC} is the weight fraction of TiC in the as-printed Al6061+TiC sample. W_{Ti} is the weight fraction of Ti (6.3%), which was determined by ICP test (the whole sample, including TiC nanoparticles, is fully dissolved during ICP test). ρ_{TiC} is the density of TiC (4930 kg m^{-3}). ρ_{nc} is the density of the Al6061+TiC, which was calculated by the following equation:

$$\rho_{\mathrm{nc}}=V_{\mathrm{TiC}}\,\rho_{\mathrm{TiC}} + (1\text{-}V_{\mathrm{TiC}})\rho_{\mathrm{Al}} \quad (2)$$

where ρ_{Al} is the density of Al6061 (2705 kg m^{-3}). Since the density of Al6061+TiC is related to the volume fraction of TiC, Equation (1) and (2) were solved iteratively until the volume fraction value stabilizes. The calculated volume fraction of TiC is 4.4%.

Supplementary Note 3: Effects of powder morphology on laser-powder interaction

Since ball-milled Al6061+4.4vol.%TiC powders introduced two variables to the Al6061 powder: altering powder morphology and adding TiC nanoparticles, we fabricated a control sample of ball-milled Al6061 powders to study the pure morphology effect. The ball milling parameter was the same as what we used for Al6061+4.4vol.%TiC powders. The powder absorptivity, melt pool dimension and large spatter generation were characterized and compared among as-received Al6061 powders, ball-milled Al6061 powders, and ball-milled Al6061+4.4vol.%TiC powders.

The measured diffuse reflectivity (measured by Perkin Elmer Lambda 19 spectrophotometer, PerkinElmer, USA) of the as-received Al6061 powders, ball-milled Al6061 powders, and ball-milled Al6061+4.4vol.%TiC powders at 1070 nm wavelength are 0.454, 0.361, 0.065, respectively (Supplementary Fig. 3a). Since the absorption length of Al and TiC at 1070 nm is small (8.2 nm and 23.3 nm, respectively, calculated based on optical constants: for TiC, n=3.96, k=3.68; for Al, n=1.37, k=10.3)[5,6], the absorptivity of the as-received Al6061 powders, ball-milled Al6061 powders, and ball-milled Al6061+4.4vol.%TiC powders were calculated as 0.546, 0.639, 0.935, respectively. Hence, changing powder morphology increased absorptivity by 17%, while simultaneously altering morphology and adding TiC nanoparticles resulted in a 71% increase in absorptivity. This suggests that adding TiC nanoparticles is the major cause of the absorptivity enhancement, which may be attributed to the high absorptivity of TiC at the wavelength of 1070 nm (absorptivity of TiC is 0.5, absorptivity of Al is 0.04)[6,7].

We further compared the melt pool dimension (transverse cross section) of the tracks printed using as-received Al6061 powders (with commercial Al6061 substrate), ball-milled Al6061 powders (with commercial Al6061 substrate), and Al6061+4.4vol.%TiC powders (with as-printed

Al6061+4.4vol.%TiC substrate) under the same processing parameter (Supplementary Fig. 3e-g). To uncover the melt pool boundary, samples were polished and lightly etched by Keller's etchant before optical observation. The melt pool widths of the as-received Al6061, ball-milled Al6061, and Al6061+4.4vol.%TiC are 406 μm, 442 μm, 579 μm, respectively. The melt pool depths of the as-received Al6061, ball-milled Al6061, and Al6061+4.4vol.%TiC are 141 μm, 145 μm, 187 μm, respectively. The melt pool width of the ball-milled Al6061 is 9% larger than that of the as-received Al6061, while the melt pool width of the Al6061+4.4vol.%TiC is 43% larger than that of the as-received Al6061. The melt pool depth of the ball-milled Al6061 is 3% larger than that of the as-received Al6061, while the melt pool depth of the Al6061+4.4vol.%TiC is 32% larger than that of the as-received Al6061. The laser power utilized during printing is 500 W. The scan speed is 0.2 m s^{-1}. The laser beam diameter (D4σ) is around 250 μm.

Additionally, the effects of powder morphology on the large spatter generation during LPBF process were studied by high-speed visible light imaging (see Method). High speed images (Supplementary Fig. 3b-d) show that many large spatters (larger than 100 μm) were generated during LPBF of the as-received Al6061 (3 ± 1 per millisecond) and ball-milled Al6061 (3 ± 1 per millisecond), but there were no large spatters for the Al6061+4.4vol.%TiC. This suggested that TiC nanoparticle is the contributor to large spatter elimination instead of powder morphology. The laser power utilized during LPBF is 500 W. The scan speed is 0.2 m s^{-1}. The laser beam diameter (D4σ) is around 250 μm. The substrates for as-received Al6061 and ball-milled Al6061 were cut from commercial Al6061 plate (T6511, Mcmaster-Carr, USA). The substrate for Al6061+4.4vol.%TiC was cut from the as-printed Al6061+4.4vol.%TiC sample.

Supplementary Note 4: Effects of surface tension and viscosity on vapor depression stability

To understand the underlying mechanism of nanoparticle-enabled stabilization of vapor depression, we measured the surface tension and viscosity of Al6061 and Al6061+4.4vol.%TiC and performed laser melting simulation (see Supplementary Note 5) with measured surface tension and viscosity value as input to study their effects on vapor depression dynamics.

The surface tension and viscosity were measured based on the oscillating droplet method[8], as schematically shown in Supplementary Fig. 7a. During the test, the sample (cut from commercial Al6061 plate or as-printed Al6061+TiC sample) was placed on top of an inert ring (which holds the droplet) with an inner diameter of 2 mm and thickness of 0.5 mm. A continuous-wave ytterbium fiber laser (IPG YLR-500-AC, IPG Photonics, USA) was used to melt the sample with a weight of 1.3×10^{-2} g corresponding to a sphere with a diameter of 2.2 mm using a laser power of 150 W, laser beam size (D4σ) of 250 μm, and heating time of 2 s. After melting, the linear solenoid was immediately triggered to accelerate the ring, which forces the liquid droplet to flow through the ring and introduces the initial deformation. The oscillation of the droplet after leaving the ring was captured by a high-speed visible light camera (FASTCAM Nova S12) at a frame rate of 10 kHz. The oscillation frequency is used to calculate the surface tension:

$$\sigma = \frac{3\pi m f^2}{8} \tag{3}$$

where σ is the surface tension, m is the mass of the droplet, f is the oscillation frequency. The damping speed of the amplitude during oscillation is used to calculate the viscosity:

$$\mu = \frac{3m}{10\pi d_0 t} \ln(\frac{\zeta_0}{\zeta}) \tag{4}$$

where μ is the viscosity, d_0 is the equilibrium diameter of the droplet, t is the oscillation time, ζ_0 is the initial amplitude, ζ is the amplitude at oscillation time t. The amplitude is determined from the vertical diameter of the droplet versus time curve in the Supplementary Fig. 7b. Based on the amplitude and frequency of the droplet oscillation, the calculated viscosity of the Al6061+4.4vol.%TiC (79.4 mPa·s) is 15 times higher than that of the Al6061 (4.9 mPa·s). The calculated surface tension of the Al6061+4.4vol.%TiC (0.81 $N \cdot m^{-1}$) is 19% higher than that of the Al6061 (0.68 $N \cdot m^{-1}$).

The measured values were then imported into simulation to study the effect of surface tension and viscosity on the vapor depression dynamics (Supplementary Fig. 8). Four simulations with different surface tension and viscosity combinations were performed (Supplementary Table 3). Other material properties and laser processing parameters (laser power of 416 W, scan speed of 0.4 m s^{-1}, laser beam diameter (D4σ) of 90 μm) were kept the same. The results show that nanoparticle-induced increase of viscosity stabilized the vapor depression (variation coefficient of vapor depression depth decreased from 0.165 to 0.092), while nanoparticle-induced increase of surface tension didn't affect vapor depression much (variation coefficient of vapor depression depth decreased from 0.165 to 0.162), as shown in Supplementary Fig. 8c, d.

Increasing viscosity stabilized the vapor depression because higher viscosity enables the viscous stress to balance the recoil pressure under smaller liquid surface deformation. During the LPBF process, the vapor depression fluctuation creates a velocity gradient along the tangential direction of the vapor depression surface, which results in the generation of viscous stress to resist further fluctuation of the vapor depression (Fig. 3k). The viscous stress is proportional to the viscosity and velocity gradient, as depicted in the following equation:

$$\tau = \mu \frac{\mathrm{d}v}{\mathrm{d}x} = \mu \frac{v_c}{h} \tag{5}$$

where τ is the viscous shear stress; μ is the viscosity; v is the moving velocity of vapor depression front wall induced by depression fluctuation, x is the distance along the vapor depression front wall, v_c is the front wall moving velocity at the center of the fluctuation, h is the distance from center to the edge of the fluctuation (Fig. 3k, l). Due to the increased viscosity (μ) by nanoparticles, smaller front wall moving velocity (v_c) is needed for generating the same viscous stress to resist recoil pressure (P_r). Therefore, the depth of the fluctuation ($d = v_c \Delta t$, where Δt is the time period and is considered constant to study the deformation within a same time period) decreased, resulting in the stabilization of vapor depression.

Supplementary Note 5: Computational thermo-fluid dynamics simulation

The vapor depression dynamics during laser melting process was simulated by FLOW-3D (FLOW-3D 12.0, Flow Sciences, USA). Throughout the simulation, the flow is assumed to be laminar and Newtonian. Governing equations include the continuity equation, momentum conservation equation and energy conservation equation:

$$\nabla\,(\rho\vec{\mathbf{v}}) = 0 \tag{6}$$

$$\frac{\partial}{\partial t}(\rho\,\vec{\mathbf{v}})+\nabla\,(\rho\,\vec{\mathbf{v}}\otimes\vec{\mathbf{v}})=\nabla\,(\mu\nabla\,\vec{\mathbf{v}})-\nabla p+\rho\vec{\mathbf{g}} \tag{7}$$

$$\frac{\partial}{\partial t}(\rho\,h)+\nabla\,(\rho\,\vec{\mathbf{v}}\,h)=q+\nabla\cdot(k\,\nabla T) \tag{8}$$

where ρ is the density, $\vec{\mathbf{v}}$ is the velocity vector, μ is the viscosity, t is the time, p is the pressure, $\vec{\mathbf{g}}$ is the gravitational acceleration vector, q is the heat source, k is the thermal conductivity, h is enthalpy, which is calculated as:

$$h=\begin{cases}\rho_s C_s T, & T\leqslant T_s\\ h(T_s)+h_{sl}\frac{T-T_s}{T_l-T_s}, & T_s<T\leqslant T_l\\ h(T_l)+\rho_l C_l(T-T_l), & T>T_l\end{cases} \tag{9}$$

where ρ_s is the density at solid state, C_s is the specific heat at solid state, T is the temperature, h_{sl} is the latent heat of melting, T_s is the solidus temperature, T_l is the liquidus temperature, ρ_l is the density at liquid state, C_l is the specific heat at liquid state.

The free surface is tracked by the Volume of Fluid (VOF) method, as denoted by the following equation:

$$\frac{\partial F}{\partial t}+\nabla\cdot(F\,\vec{\mathbf{v}})=0 \tag{10}$$

where F is the phase fraction. Thus, fluid exists within regions of 'F = 1', whilst 'F = 0' corresponds to regions considered as voids with uniform pressure. The interface reconstruction is carried out using the split Lagrangian scheme.

The multiple reflection model based on ray-tracing technique is implemented in the simulation. For each incidence, and absorption is calculated by the Fresnel equation:

$$A=1-\frac{1}{2}\left(\frac{1+(1-\varepsilon\cos\theta)^2}{1+(1+\varepsilon\cos\theta)^2}+\frac{\varepsilon^2-2\varepsilon\cos\theta+2\cos^2\theta}{\varepsilon^2+2\varepsilon\cos\theta+2\cos^2\theta}\right) \quad (11)$$

where θ is the incident angle; ε is a constant related to the material and laser properties, which was calibrated by experimental data captured by x-ray imaging (length and depth of melt pool, depth of vapor depression).

To study the vapor depression dynamics, the recoil pressure, which is the major driving force for vapor depression formation, is considered based on the following equation:

$$P_r=0.54P_0\exp\left[-\frac{\lambda}{K_B}\left(\frac{1}{T}-\frac{1}{T_b}\right)\right] \quad (12)$$

where P_0 is the ambient pressure, λ is the latent heat of vaporization, K_B is the Boltzmann constant, T is the surface temperature, T_b is the boiling temperature. Other driving forces including thermocapillary force, gravity force and buoyancy force are also considered in the model. The properties of Al6061 used in simulation are presented in Supplementary Table 4. The dimension of the Al6061 plate in simulation is 3 mm (length) × 0.7 mm (width) × 0.5 mm (depth) with an initial temperature of 298 K. The mesh size is 4 µm.

Supplementary Note 6: Guideline for estimating nanoparticle volume fraction needed for controlling spatter coalescence

To control spatter coalescence and eliminate large spatters, we proposed that nanoparticles should form a close packing on metal powder/spatter surface. If nanoparticles are not closely packed, it is likely to generate an exposed surface area without nanoparticles (Supplementary Fig. 10b). If two exposed surfaces come into contact during spatter colliding, the spatters can easily coalesce. This is also confirmed by the capillary pressure model, based on which the effects of interparticle spacing on the capillary pressure were calculated (assuming contact angle of 52°). The results (Supplementary Fig. 10d) show that there is a sudden drop of capillary pressure barrier when the interparticle distance starts to increase (capillary pressure decreases by 51% when the ratio of interparticle distance to nanoparticle diameter increases from 1.05 to 3). This will increase the possibility of spatter coalescence.

If only one spatter has closely packed nanoparticles, while another one has exposed surface area (Supplementary Fig. 10c), there will be only one-layer nanoparticle between two liquid spatters. The capillary pressure barrier generated by one-layer nanoparticle (based on capillary pressure model) is significantly weaker than that generated by two layers (Supplementary Fig. 10e). For example, the highest temperature at which the capillary pressure overcomes the initial pressure decreases from 1420 K for two-layer nanoparticle to 1159 K for one-layer nanoparticle. Therefore, for all spatters, the one-layer close packing of nanoparticles on the surface is required.

To guide the material design, we developed an equation to calculate the volume fraction of nanoparticles needed for close packing based on the relationship between nanoparticle cross-section area and metal powder surface area:

$$f \cdot N_m \cdot A_m = N_n \cdot A_n \tag{13}$$

where f is the nanoparticle surface converging fraction (the projection areas of the nanoparticles to the surface area of the spatter) for close packing (here assumed $f = 0.907$ for hexagonal close packing). N_m is the number of metal powders, A_m is the surface area of metal powder, N_n is the number of nanoparticles, A_n is the cross-section area of nanoparticle. The number of metal powder/ceramic particle (N_m, N_n) in Equation (13) can be calculated by dividing the total volume by the volume of single metal powder/ceramic particle. Therefore, Equation (13) can be written as:

$$f \cdot [(1-V)/(4\pi r_m^3/3)] \cdot 4\pi r_m^2 = [V/(4\pi r_n^3/3)] \cdot \pi r_n^2 \tag{14}$$

V is the volume fraction of nanoparticles. r_m is the radius of metal powder; r_n is the radius of nanoparticle. Assuming metal powder size is much larger than nanoparticle size, based on equation (14), the volume fraction of nanoparticles can be expressed as:

$$V = 3.6 r_n / r_m \tag{15}$$

It should be noted that given the nanoparticles may be not uniformly dispersed on the metal powder surface, the calculated volume fraction of nanoparticles is a minimum number.

Supplementary Note 7: Estimation of the inertial pressure of liquid spatter

The inertial pressure (p_i) of liquid spatter arises from the momentum of the flying spatter and was calculated as[9]:

$$p_i = \frac{\rho v^2}{2} \tag{16}$$

where ρ is the density of liquid Al6061 (2415 kg m^{-3})[1], v is the largest spatter flying velocity captured by x-ray imaging (6.9 m s^{-1}). If the inertial pressure is high enough to overcome capillary pressure barrier, two spatters will coalesce.

Supplementary References

1. Mills, K. C. *Recommended values of thermophysical properties for selected commercial alloys*. (Woodhead Publishing, 2002).

2. Karnati, S., Axelsen, I., Liou, F. F. & Newkirk, J. W. Investigation of tensile properties of bulk and SLM fabricated 304L stainless steel using various gage length specimens. in *Proceedings of the 27th Annual International Solid Freeform Fabrication Symposium–An Additive Manufacturing Conference* 592–604 (2016).

3. Lin, T. C. *et al.* Aluminum with dispersed nanoparticles by laser additive manufacturing. *Nat. Commun.* **10**, 4124 (2019).

4. Karantzalis, A. E., Lekatou, A., Georgatis, M., Poulas, V. & Mavros, H. Casting-based production of Al-TiC-AlB_2 composite material through the use of KBF_4 salt. *J. Mater. Eng. Perform.* **20**, 198–202 (2011).

5. Lynch, D. W., Olson, C. G., Peterman, D. J. & Weaver, J. H. Optical properties of $TiC_x(0.64 \le x \le 0.90)$ from 0.1 to 30 eV. *Phys. Rev. B* **22**, 3991 (1980).

6. Rakić, A. D. Algorithm for the determination of intrinsic optical constants of metal films: application to aluminum. *Appl. Opt.* **34**, 4755 (1995).

7. Karlsson, B., Sundgren, J. E. & Johansson, B. O. Optical constants and spectral selectivity of titanium carbonitrides. *Thin Solid Films* **87**, 181–187 (1982).

8. Ma, C., Zhao, J., Cao, C., Lin, T. & Li, X. Fundamental study on laser interactions with nanoparticles-reinforced metals—part II: effect of nanoparticles on surface tension, viscosity, and laser melting. *J. Manuf. Sci. Eng.* **138**, 121002 (2016).

9. Khairallah, S. A. *et al.* Controlling interdependent meso-nanosecond dynamics and defect generation in metal 3D printing. *Science* **368**, 660–665 (2020).
