# Supplementary material for: Controlling process instability for defect lean metal additive manufacturing
Source: Nat Commun. 2022 Feb 28;13:1079. doi: 10.1038/s41467-022-28649-2 (PMC8885710; doi:10.1038/s41467-022-28649-2)
Supplement: Supplementary file 2 — Description of Additional Supplementary Files [file 41467_2022_28649_MOESM2_ESM.pdf]

Description of Additional Supplementary Files

File Name: Supplementary Movie 1
Description: Spatter behavior comparison of Al6061 and Al6061+4.4vol.%TiC under the same processing parameter. Large spatters (defined as the spatter with the size larger than 100 μm) were eliminated during LPBF of Al6061+4.4vol.%TiC. The laser power is 364 W and the scan speed is 0.4 m s^{-1}.

File Name: Supplementary Movie 2
Description: Spatter behavior comparison of Al6061 and Al6061+4.4vol.%TiC under conduction mode. Large spatters were eliminated during LPBF of Al6061+4.4vol.%TiC. The laser power is 312 W for Al6061 and 208 W for Al6061+4.4vol.%TiC. The scan speed is 0.4 m s^{-1} for both.

File Name: Supplementary Movie 3
Description: Spatter behavior comparison of Al6061 and Al6061+4.4vol.%TiC under keyhole mode. Large spatters were eliminated during LPBF of Al6061+4.4vol.%TiC. The laser power is 416 W for Al6061 and 312 W for Al6061+4.4vol.%TiC. The scan speed is 0.4 m s^{-1} for both.

File Name: Supplementary Movie 4
Description: Nanoparticle-enabled elimination of liquid breakup during LPBF process. Liquid breakup was observed during LPBF of Al6061. No liquid breakup was observed during LPBF of Al6061+4.4vol.%TiC. The laser power is 416 W for Al6061 and 312 W for Al6061+4.4vol.%TiC. The scan speed is 0.4 m s^{-1} for both.

File Name: Supplementary Movie 5
Description: Dynamics of spatter agglomeration during LPBF of Al6061. The laser power is 312 W. The scan speed is 0.4 m s^{-1}.

File Name: Supplementary Movie 6
Description: Nanoparticle-enabled prevention of spatter coalescence during colliding in Al6061+4.4vol.%TiC. The laser power is 312 W. The scan speed is 0.4 m s^{-1}.
